# Use of hospital services by patients with chronic conditions in sub-Saharan Africa: a systematic review and meta-analysis

**DOI:** 10.2471/BLT.22.289597

**Published:** 2023-07-05

**Authors:** Stephen A Spencer, Jamie Rylance, Jennifer K Quint, Stephen B Gordon, Paul Dark, Ben Morton

**Affiliations:** a Department of Clinical Sciences, Liverpool School of Tropical Medicine, Pembroke Place, Liverpool, L3 5QA, England.; b National Heart and Lung Institute, Imperial College London, London, England.; c Malawi-Liverpool-Wellcome Trust Clinical Research Programme, Blantyre, Malawi.; d Humanitarian and Conflict Response Institute, University of Manchester, Manchester, England.

## Abstract

**Objective:**

To estimate the prevalence of individual chronic conditions and multimorbidity among adults admitted to hospital in countries in sub–Saharan Africa.

**Methods:**

We systematically searched MEDLINE®, Embase®, Global Index Medicus, Global Health and SciELO for publications reporting on patient cohorts recruited between 1 January 2010 and 12 May 2023. We included articles reporting prevalence of pre-specified chronic diseases within unselected acute care services (emergency departments or medical inpatient settings). No language restrictions were applied. We generated prevalence estimates using random-effects meta-analysis alongside 95% confidence intervals, 95% prediction intervals and *I^2^* statistics for heterogeneity. To explore associations with age, sex, country-level income status, geographical region and risk of bias, we conducted pre-specified meta-regression, sub-group and sensitivity analyses.

**Findings:**

Of 6976 identified studies, 61 met the inclusion criteria, comprising data from 20 countries and 376 676 people. None directly reported multimorbidity, but instead reported prevalence for individual conditions. Among medical admissions, the highest prevalence was human immunodeficiency virus infection (36.4%; 95% CI: 31.3–41.8); hypertension (24.4%; 95% CI: 16.7–34.2); diabetes (11.9%; 95% CI: 9.9–14.3); heart failure (8.2%; 95% CI: 5.6–11.9); chronic kidney disease (7.7%; 95% CI: 3.9–14.7); and stroke (6.8%; 95% CI: 4.7–9.6).

**Conclusion:**

Among patients seeking hospital care in sub-Saharan Africa, multimorbidity remains poorly described despite high burdens of individual chronic diseases. Prospective public health studies of multimorbidity burden are needed to generate integrated and context-specific health system interventions that act to maximize patient survival and well-being.

## Résumé

**Objectif:**

Mesurer le taux de prévalence des maladies chroniques individuelles et de la multimorbidité chez les adultes hospitalisés en Afrique subsaharienne.

**Méthodes:**

Nous avons analysé systématiquement MEDLINE®, Embase®, l'Index Medicus mondial, Global Health et SciELO à la recherche de publications mentionnant des cohortes de patients recrutés entre le 1^er^ janvier 2010 et le 12 mai 2023. Nous avons retenu les articles traitant de la prévalence de certaines maladies chroniques prédéfinies au sein des services de soins intensifs, sans sélection préalable (aux urgences ou en milieu hospitalier). Aucune restriction de langue n'a été appliquée. Nous avons ensuite estimé le taux de prévalence au moyen d'une méta-analyse à effets aléatoires, tout en appliquant des intervalles de confiance de 95%, des intervalles de prévision de 95% et une statistique *I^2^* pour l'hétérogénéité. Enfin, en vue d'examiner les liens avec l'âge, le sexe, le statut de revenu du pays, la situation géographique et le risque de biais, nous avons mené des analyses prédéfinies de méta-régression, de sous-groupe et de sensibilité.

**Résultats:**

Sur les 6976 études identifiées, 61 correspondaient aux critères d'inclusion et contenaient des informations relatives à 20 pays et 376 676 personnes. Bien qu'aucune n'évoquait directement une multimorbidité, la prévalence des cas individuels était indiquée. Parmi les admissions à l'hôpital, le plus haut taux de prévalence concernait: les infections au virus de l'immunodéficience humaine (36,4%; IC de 95%: 31,3–41,8), l'hypertension (24,4%; IC de 95%: 16,7–34.2), le diabète (11,9%; IC de 95%: 9,9–14,3), l'insuffisance cardiaque (8,2%; IC de 95%: 5,6–11,9), les maladies rénales chroniques (7,7%; IC de 95%: 3,9–14,7) et les accidents vasculaires cérébraux (6,8%; IC de 95%: 4,7–9,6).

**Conclusion:**

La multimorbidité des patients se rendant à l'hôpital en Afrique subsaharienne demeure peu documentée, en dépit de la lourde charge que font peser les maladies chroniques individuelles. Des études prospectives de santé publique sont nécessaires à ce sujet pour déclencher des interventions du système de santé adaptées au contexte et améliorer ainsi les chances de survie et le bien-être des patients.

## Resumen

**Objetivo:**

Estimar la prevalencia de enfermedades crónicas individuales y la multimorbilidad en adultos ingresados en hospitales en países del África Subsahariana.

**Métodos:**

Buscamos publicaciones de manera sistemática en MEDLINE®, Embase®, Global Index Medicus, Global Health y SciELO que registraran grupos de pacientes reclutados entre el 1 de enero de 2010 y el 12 de mayo de 2023. Incluimos artículos que informaban sobre la prevalencia de enfermedades crónicas predefinidas en el marco de los servicios de cuidados intensivos no seleccionados (salas de urgencias o centros médicos hospitalarios). No se aplicaron restricciones lingüísticas. Generamos estimaciones sobre la prevalencia utilizando un metaanálisis de efectos aleatorios, junto con intervalos de confianza del 95%, intervalos de predicción del 95% y estadística *I^2^* de heterogeneidad. Para analizar la relación con la edad, el sexo, el nivel de ingresos del país, la zona geográfica y el riesgo de sesgo, llevamos a cabo una metarregresión predefinida y un análisis de subgrupos y sensibilidad.

**Resultados:**

De los 6976 estudios seleccionados, 61 cumplían los criterios de inclusión, y englobaban datos de 20 países y 376 676 personas. Ninguno registraba multimorbilidad de manera directa, sino prevalencia de enfermedades individuales. Entre los ingresos médicos, la prevalencia más alta correspondió a: la infección del virus de la inmunodeficiencia humana (36,4%; IC del 95%: 31,3–41,8), hipertensión (24,4%; IC del 95%: 16,7–34,2), diabetes (11,9%; IC del 95%: 9,9–14,3), insuficiencia cardíaca (8,2%; IC del 95%: 5,6–11,9), enfermedad crónica renal (7,7%; IC del 95%: 3,9–14,7) e ictus (6,8%; IC del 95%: 4,7–9,6).

**Conclusión:**

Entre los pacientes que buscan atención hospitalaria en el África Subsahariana, la multimorbilidad sigue estando escasamente descrita, a pesar de las elevadas tasas de enfermedades crónicas individuales. Los estudios prospectivos de salud pública que versan sobre tasas de multimorbilidad son necesarios para generar intervenciones integradas y adaptadas a las circunstancias por parte del sistema sanitario, para poder aumentar así la supervivencia y el bienestar de los pacientes.

## ملخص

**الغرض:**

تقدير انتشار الحالات الفردية المزمنة وتعدد الأمراض بين البالغين الذين يدخلون المستشفيات في جنوب الصحراء الكبرى بأفريقيا.

**الطريقة:**

قمنا بالبحث المنهجي في MEDLINE®‎، وEmbase®‎، وGlobal Index Medicus، وGlobal Health، وSciELO عن المنشورات التي تقدم تقارير عن مجموعات المرضى المعينين بين 1 يناير/كانون ثاني 2010، و12 مايو/أيار 2023. لقد قمنا بتضمين المقالات التي تشير إلى انتشار الأمراض المزمنة المحددة مسبقًا ضمن خدمات الرعاية الدقيقة غير المختارة (أقسام الطوارئ أو الأوضاع الطبية لمرضى العيادات الداخلية). لم يتم تطبيق أية قيود لغوية. لقد أنشأنا تقديرات الانتشار باستخدام التحليل التلوي للتأثيرات العشوائية جنبًا إلى جنب مع فواصل ثقة مقدارها %95، وفواصل للتنبؤ مقدارها %95، وإحصاءات *I *^2 ^ عدم التجانس. لاستكشاف الارتباطات بالعمر، والجنس، وحالة الدخل على مستوى الدولة، والمنطقة الجغرافية، وخطر التحيز، قمنا بإجراء تحليلات التحوف التلوي، والمجموعة الفرعية، والحساسية، المحددة مسبقًا.

**النتائج:**

من بين 6976 دراسة تم تحديدها، استوفت 61 دراسة منها معايير الاشتمال، وتضمنت بيانات من 20 دولة، و376676 شخصًا. لم يُبلغ أي منها بشكل مباشر عن تعدد الأمراض، ولكنها أبلغت بدلاً من ذلك عن انتشار الحالات الفردية. من بين الحالات الطبية للالتحاق بالمستشفى، كان أعلى معدل انتشار هو: الإصابة بفيروس اضطراب نقص المناعة البشرية (%36.4؛ بفاصل ثقة مقداره %95: 31.3 إلى 41.8)، وارتفاع ضغط الدم (%24.4؛ بفاصل ثقة مقداره %95: 16.7 إلى34.2)، ومرض السكري (%11.9؛ بفاصل ثقة مقداره %95: 9.9 إلى 14.3)، وقصور القلب (%8.2؛ بفاصل ثقة مقداره %95: 5.6 إلى 11.9)، ومرض الكلى المزمن (%7.7؛ بفاصل ثقة مقداره %95: 3.9 إلى 14.7)، والسكتة الدماغية (%6.8؛ بفاصل ثقة مقداره %95: 4.7 إلى 9.6).

**الاستنتاج:**

يظل تعدد الأمراض فقير الوصف على الرغم من الأعباء الكبيرة للأمراض المزمنة الفردية، بين المرضى الذين يسعون للحصول على الرعاية في المستشفيات في جنوب الصحراء الكبرى بأفريقيا. هناك حاجة لدراسات الصحة العامة المستقبلية لعبء تعدد الأمراض، وذلك لتجهيز تدخلات نظام صحي متكامل ومحددة السياق، تعمل على زيادة فرص حياة المريض وعافيته.

## 摘要

**目的:**

旨在评估撒哈拉以南非洲地区住院的成年患者中个体慢性病和共病的患病率。

**方法:**

我们系统地检索了 MEDLINE®、Embase®、全球医学索引、全球健康 和 SciELO 中的数据，以获取关于在 2010 年 1 月 1 日至 2023 年 5 月 12 日期间报告招募患者群的论文。我们纳入了在未经选择的急症护理服务（急诊科或医疗住院场所）中报告预先指定的慢性疾病患病率的文章。没有语言限制。我们使用随机效应 meta 分析以及 95% 置信区间、95% 预测区间和 I2异质性统计量生成患病率估计值。为了探索与年龄、性别、国家收入状况、地理区域和偏倚风险的相关性，我们进行了预先指定的 meta 回归、亚组和敏感性分析。

**结果:**

在 6,976 项识别出的研究中，有 61 项符合纳入标准，包括来自 20 个国家和 376,676 名患者的数据。没有研究直接报告共病，都是报告个体疾病的患病情况。在住院患者中，患病率最高的是：人类免疫缺陷病毒感染（36.4%；95% CI：31.3-41.8），高血压（24.4%；95% CI：16.7–34.2），糖尿病（11.9%；95% CI：9.9-14.3），心力衰竭（8.2%；95% CI：5.6-11.9），慢性肾脏病（7.7%；95% CI：3.9-14.7）以及中风（6.8%；95% CI：4.7-9.6）。

**结论:**

在撒哈拉以南非洲地区寻求医院治疗的患者中，尽管个体慢性疾病治疗负担沉重，但对共病的报告仍然很少。需要对共病治疗负担进行前瞻性公共卫生研究，以制定综合和针对具体情况的卫生系统干预措施，最大限度地提高患者的生存率和福祉。

## Резюме

**Цель:**

Оценить распространенность отдельных хронических заболеваний и мультиморбидности среди взрослого населения, поступившего в стационар в странах Африки к югу от Сахары.

**Методы:**

Проведен систематический поиск в MEDLINE®, Embase®, Global Index Medicus, Global Health и SciELO публикаций, содержащих сведения о когортах пациентов, набранных в период с 1 января 2010 г. по 12 мая 2023 г. Включены статьи, в которых сообщалось о распространенности определенных хронических заболеваний в невыборочных службах неотложной помощи (отделениях скорой помощи или медицинских стационарах). Языковые ограничения отсутствовали. Были получены оценки распространенности с помощью метаанализа со случайными эффектами, а также 95%-е доверительные интервалы, 95%-е интервалы предсказания и статистика *I^2^* для оценки гетерогенности. Для изучения ассоциаций с возрастом, полом, уровнем дохода в стране, географическим регионом и риском систематической ошибки авторы провели заранее оговоренные анализы метарегрессии, подгрупп и чувствительности.

**Результаты:**

Из 6976 выявленных исследований 61 отвечало критериям включения и содержало данные из 20 стран и 376 676 человек. Ни в одном из них не было прямого указания на мультиморбидность, а вместо этого приводились данные о распространенности отдельных заболеваний. Среди госпитализаций наибольшую распространенность имели вирусная инфекция иммунодефицита человека (36,4%; 95%-й ДИ: 31,3–41,8), гипертония (24,4%; 95%-й ДИ:16,7–34,2), диабет (11,9%; 95%-й ДИ: 9,9–14,3), сердечная недостаточность (8,2%; 95%-й ДИ: 5,6–11,9), хроническая болезнь почек (7,7%; 95%-й ДИ: 3,9–14,7) и инсульт (6,8%; 95%-й ДИ: 4,7–9,6).

**Вывод:**

Среди пациентов, обращающихся за стационарной медицинской помощью в странах Африки к югу от Сахары, мультиморбидность остается малоописанной, несмотря на высокое бремя отдельных хронических заболеваний. Проспективные исследования общественного здравоохранения по изучению мультиморбидности необходимы для разработки комплексных и учитывающих конкретные условия вмешательств в систему здравоохранения, направленных на максимальное увеличение выживаемости и благополучия пациентов.

## Introduction

As life expectancy increases in sub-Saharan Africa, so too does the number of people who live with chronic conditions. Multimorbidity is defined as living with two or more chronic health conditions, inclusive of interactions between chronic communicable diseases and noncommunicable diseases.^1^^–^^3^ Inequalities in access to health care for chronic conditions affect early detection and control, and therefore on healthy life expectancy. Where primary care provision is limited, the index presentation of chronic disease is commonly through hospital acute care services.^4^^,^^5^ Acute medical services in these contexts traditionally have a single disease focus and may overlook multimorbidity in vulnerable patients.

In sub-Saharan Africa, the burden from chronic diseases is projected to increase: an estimated 125 million adults will have hypertension by 2025;^6^ and 26.9 million adults will have diabetes by 2030.^7^ Although dramatic reductions in the incidence and mortality of human immunodeficiency virus (HIV) have been observed in sub-Saharan Africa over the past 30 years, with increasing life expectancy, the high prevalence of HIV infection is presenting new challenges and demands within existing health-care systems.^8^ As such, integration of multimorbidity care into hospitals in sub-Saharan Africa will be of increasing importance over coming years. Cohort studies of adults in community settings have reported prevalence of multimorbidity of 69% (absolute numbers not available) in South Africa and 65% (252/389) in Burkina Faso.^2^^,^^9^ However, data on the prevalence of individual chronic diseases and multimorbidity in sub-Saharan African hospital settings are limited.^10^

To estimate prevalence of chronic disease within unselected cohorts of adult patients admitted to medical wards and emergency departments within sub-Saharan Africa, we conducted a systematic review of observational epidemiological studies. We focused on hospital rather than community presentations as populations in sub-Saharan Africa commonly have limited access to primary care. As such, hospital presentation represents an important node of intervention to control chronic disease and prevent development of secondary complications. Development of prevalence estimates within the region are important for policy-makers to prioritize and optimize service design and care delivery in sub-Saharan Africa.^9^^,^^11^

## Methods

We conducted and reported this PROSPERO-registered systematic review (ID: CRD42021262708) in line with the PRISMA 2020 statement.^12^

### Eligibility criteria

We employed the condition, context and population strategy (Box 1) to define our study population, in line with guidance for systematic reviews of observational epidemiological studies reporting prevalence data.^13^ Inclusion criteria were studies on adults in sub-Saharan Africa who had an acute hospital admission to emergency department or medical ward (representative of an unselected inpatient population in either emergency departments or medical wards). Data on outcome conditions are available (Box 2).

Box 1 **Conditions, context, population criteria and search strategy used for the systematic review on patients with chronic conditions in sub-Saharan Africa**

**Criteria**

Conditions: Chronic diseases or risk factors that are likely to contribute to multimorbidity.

Context: Acute admission to adult medical wards or emergency departments in hospitals in sub-Saharan Africa.

Population: Adults of both sexes that meet the ‘context’ criteria (above).

**Search strategy**

(Hypertension OR diabetes OR obesity OR alcohol use OR tobacco OR kidney dysfunction OR hypercholesterolaemia OR HIV/AIDS OR HIV treatment failure OR HIV treatment non-compliance OR stroke OR ischaemic heart disease OR chronic liver disease OR heart failure OR chronic kidney disease OR chronic obstructive pulmonary disease OR multimorbidity) AND sub-Saharan Africa AND acute hospital care AND adults

Box 2 **Outcome conditions used for the systematic review on patients with chronic conditions in sub-Saharan Africa**

We identified chronic conditions contributing to potential multimorbidity in adults in sub-Saharan Africa from the Global Burden of Disease 2019 databases for risks (risk factor conditions) and causes (diseases).^14^ The top 15 common causes resulting in death were included. In addition, HIV treatment failure and non-compliance were included a priori as significant drivers of HIV morbidity and mortality.^15^

**Primary outcomes**

Prevalence of the pre-selected primary preventive conditions and secondary (end-organ) conditions

**Primary preventive conditions:**

HIV, hypertension, diabetes, obesity, alcohol use, smoking and dyslipidaemia

**Secondary (end-organ) conditions:**

Stroke, ischaemic heart disease (including acute coronary syndrome), heart failure, chronic liver disease, chronic kidney disease and chronic obstructive pulmonary disease (COPD)

Secondary outcomes:

(i) prevalence of multimorbidity in acutely unwell adult patients presenting to hospitals in sub-Saharan Africa; (ii) prevalence of decompensated chronic disease-associated admission; and (iii) prevalence of HIV treatment failure, HIV treatment non-compliance, undiagnosed HIV and HIV status awareness.

HIV: human immunodeficiency virus.

Exclusion criteria were paediatric populations; community or out-patient settings (not acute care); denominator not available for population of interest (e.g. selected disease-specific cohorts or patients recruited solely from renal or cardiology wards); mental health conditions; trauma or surgical conditions; maternal, obstetric or gynaecological conditions; behavioural risk factors (excluding alcohol and tobacco); conference abstracts. 

We excluded paediatric populations as patterns and clustering of multimorbidity in children younger than five years has been reviewed elsewhere, and found to be different than adult populations.^16^ Similarly, multimorbidity in maternal care in sub-Saharan Africa has recently been examined, suggesting a specific analysis for non-pregnant adults would complement these efforts.^17^ We restricted studies to those published and conducted since 1 January 2010 to avoid the use of data before the accelerated roll-out of antiretroviral treatment (ART) in sub-Saharan Africa which has driven changes in disease patterns.^18^ The 2010 cut-off is aligned with reporting frames of the Joint United Nations Programme on HIV/AIDS (UNAIDS) and Global Burden of Disease (GBD) studies.^8^^,^^18^^,^^19^ We did not apply language restrictions to inclusion criteria.

### Databases and search terms

We systematically searched MEDLINE®, Embase®, Global Index Medicus, Global Health and SciELO databases on 12 May 2023 for articles published since 1 January 2010. EndNote X9.3.3 software (Thomson Reuters, Eagan, United States of America) was used to export references, and to identify and remove duplicates. Box 1 shows key search terms; the full search strategy is available in the online data repository.^20^

### Selection process and data collection

Two authors independently assessed article titles, abstracts and full manuscripts to select studies meeting the eligibility criteria. Subsequently they piloted and refined the data collection tool^20^ using the first five eligible studies. These two authors then independently and manually extracted data from each manuscript, and assessed for bias using the modified Newcastle-Ottawa Scale for non-randomized studies^21^ (online repository).^20^ We categorized scores of ≤ 3 as very high risk of bias; 3–6 as high risk of bias; and scores of 7–9 as high quality.^22^ Discrepancies in selection and bias decisions were resolved through discussion and arbitration by a third reviewer.

### Analysis

We captured extracted data using Microsoft Excel (Microsoft Corporation, Redmond, USA) and analysed using Stata 15 (StataCorp LLC, College Station, USA). We assessed publication bias by visual inspection of funnel plots of prevalence data when > 10 prevalence estimates were included,^23^ and Egger’s test.^24^ To visualize and assess individual disease prevalence alongside both 95% confidence and prediction levels, we generated forest plots with meta-analyses.^25^ We chose random effects modelling a priori due to the expected high level of heterogeneity,^26^ and we calculated pooled confidence with heterogeneity by the Hartung-Knapp-Sidik-Jonkman method.^27^^,^^28^ Data were logit transformed except when close to the extreme boundaries, where Freeman-Tukey double-arcsine transformation^29^ was employed (the full STATA statistical analysis code is available in the online repository).^20^

Heterogeneity was assessed by *I^2^* statistic and by 95% prediction intervals (95% PI) which estimate the range of values in which future similar studies would be expected to fall.^30^ Random effects models were used to calculate 95% PIs when ≥ 5 study estimates were included in the meta-analysis, due to the high degree of imprecision with very low numbers of estimates.^31^^,^^32^

We performed meta-regression analysis where > 10 prevalence estimates were present per condition.^33^ We included a priori within univariable meta-regression analyses: age (median or mean); sex; date of study. In view of continued expansion in ART availability across sub-Saharan Africa,^34^ we also examined the temporal changes in the prevalence of conditions through meta regression. We also used meta regression to assess the association between study-level HIV prevalence and country-level adult HIV prevalence (as given by GBD 2019 database for adults ≥ 20 years).^14^

We pre-planned to report all prevalence estimates stratified by hospital population (medical vs emergency department); country-level income status defined by the World Bank 2022 Fiscal Year;^35^ geographical regions defined by the African Union (Central, Eastern, Southern, and Western Africa);^36^ and Newcastle-Ottawa-Scale. We also planned in advance to report prevalence estimates among the high- and mid-quality graded studies through sensitivity analyses once studies with a very high risk of bias were removed.

## Results

**Fig. 1:**
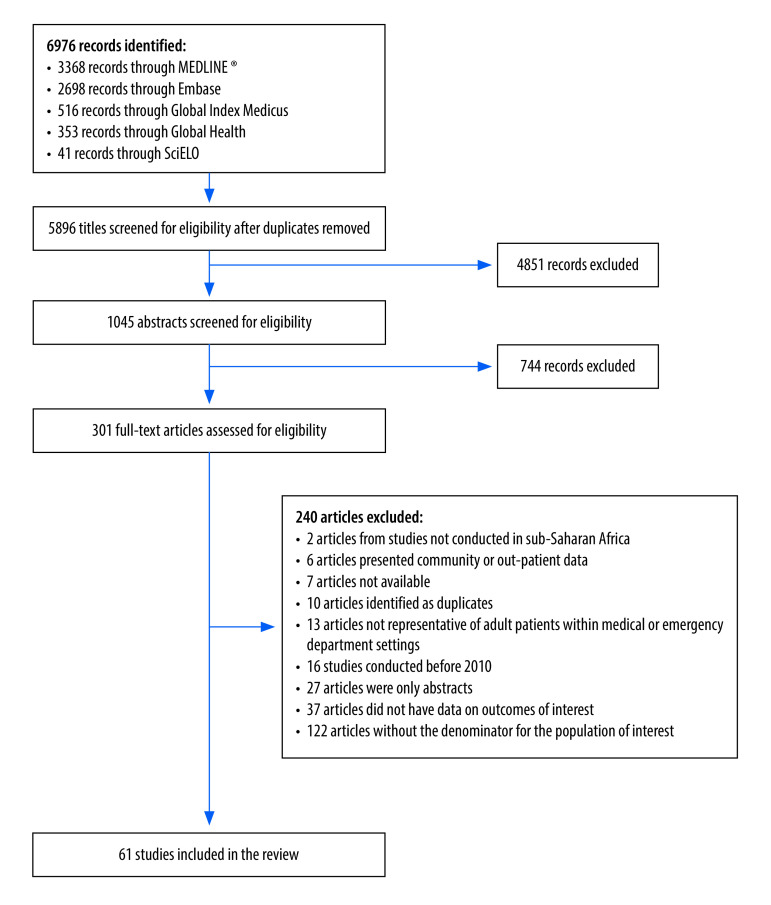
Flowchart showing the selection of studies included in the systematic review on patients with chronic conditions in sub-Saharan Africa

We identified 6976 manuscripts, of which 61 studies met the inclusion criteria (Fig. 1). These articles included 17 prospective cohort studies;^37^^–^^53^ 11 retrospective cohorts;^54^^–^^64^ and 33 cross-sectional studies.^65^^–^^97^ The pooled sample size was 376 676 participants, including 97 737 participants admitted to the emergency department, and 278 939 admitted to medical wards. We did not identify any studies that intentionally investigated prevalence of multimorbidity as a primary objective. We have therefore structured the results section to explore the prevalence of the most commonly identified individual chronic diseases, followed by a section exploring available data on multimorbidity from secondary analyses of included studies.

Characteristics of studies that met the inclusion criteria are described in Table 1 (available at: https://www.who.int/publications/journals/bulletin/). Data derived from 20 of the 48 countries in sub-Saharan Africa, predominantly from South Africa (10/61; 16.4%), United Republic of Tanzania (7/61; 11.5%) and Ethiopia (7/61; 11.5%). Forty-two studies (68.9%) examined unselected patients on medical wards and 19 (31.2%) studies examined unselected patients from the emergency department. Studies were assessed as high quality in 27 (44.3%) cases, with high risk of bias in 26 (42.6%), and very high risk of bias in eight (13.1%; online repository).^20^

**Table 1:** Included studies in the systematic review on patients with chronic conditions, sub-Saharan Africa

Study	Country	Data collection year	Site	Study design	Outcome and outcome assessment	Sample size	Age of patients, years	No. of female patients (%)
Sheikh Hassan et al., 2023^93^	Somalia	2021–2022	Emergency department	Cross sectional	Stroke. Neurologist confirmed diagnosis based on clinical symptoms, signs, CT, MRI, EEG and laboratory results	8500	NR	NR
Sendekie et al., 2023^53^	Ethiopia	2022	Medical ward	Prospective cohort	Stroke, heart failure, diabetes mellitus, chronic kidney disease and chronic liver disease. All diagnoses ascertained from patient notes	237	Mean: 53 (SD: 18)	114 (48.1)
Ronny et al., 2022^52^	Uganda	2020	Medical ward	Prospective cohort	Hypertension, HIV, diabetes mellitus, smoking and renal impairment. Renal impairment: diagnosed using urinalysis and/or estimated glomerular filtration rate. Other diagnoses ascertained from medical history.	357	Median: 47 (IQR: 32–63)	168 (47.1)
Musung et al., 2022^91^	Democratic Republic of the Congo	2018–2020	Medical ward	Cross sectional	Stroke. Clinical presentation of stroke confirmed by CT scan	9919	NR	NR
Kemal et al., 2022^94^	Ethiopia	2022	Medical ward	Cross sectional	Stroke, diabetic emergencies and HIV status not known (at discharge). All diagnoses ascertained from medical notes	423	Median: 45	217 (51.3)
Kazibwe et al., 2022^64^	Uganda	2011–2019	Medical ward	Retrospective cohort study	HIV, diabetes mellitus and hypertension. Clinical diagnoses extracted from notes, based on history, examination and laboratory findings	108 357	Mean: 43 (SD: 19)	55 620 (51.3)
Ibrahim et al., 2022^54^	Nigeria	2015–2019	Emergency department	Retrospective cohort	Stroke. Clinical presentation of stroke, confirmed by CT scan	5944	NR	NR
Roberts et al., 2021^65^	South Africa	2018	Emergency department	Cross sectional	HIV, HIV new diagnosis and HIV status not known (at discharge). Point-of-care HIV test	790	NR	431 (54.6)
Iradukunda et al., 2021^66^	Burundi	2019	Medical ward	Cross sectional	Hypertension, diabetes mellitus, alcohol use and smoking. Hypertension: SBP > 140 mmHg and/or DBP > 90 mmHg after three readings	353	NR	147 (41.6)
Ephraim et al., 2021^67^	Ghana	2017–2018	Medical ward	Cross sectional	Hypertension, alcohol use, smoking and renal impairment. Hypertension: duplicate blood pressure readings after 5 minutes rest. Renal impairment: creatinine at admission and 48 hours. Kidney disease criteria^a^	76	Mean: 47 (SD: 18)	45 (59.2)
Pintye et al., 2021^68^	Botswana	2018–2019	Emergency department	Cross sectional	HIV new diagnosis. HIV: HIV testing conducted per national guidelines	9695	Median: 30 (IQR 23–41)	4953 (51.1)
Mouton et al., 2021^69^	South Africa	2014–2015	Medical ward^ b^	Cross sectional	Hypertension, HIV, diabetes mellitus, heart failure, chronic kidney disease and chronic obstructive pulmonary disease. All diagnoses ascertained from medical notes	1010	Median: 44 (IQR: 31–61)	580 (57.4)
Moretti et al., 2021^55^	Rwanda	2013–2016	Medical ward^ b^	Retrospective cohort	Hypertension, HIV, stroke, chronic liver disease, heart failure, chronic kidney disease and renal impairment. All diagnoses ascertained from medical notes and vital signs	1704	Median: 41 (IQR: 29–59)	795 (57.4)
Mkoko et al., 2021^70^	South Africa	2016	Medical ward	Cross sectional	Hypertension, Hypertension emergency, HIV, stroke, acute coronary syndrome and diabetes mellitus. Diagnoses based on medical records, available imaging and laboratory results. Diagnosis made by qualified physicians	4884	NR	2727 (55.8)
Laher et al., 2021^37^	South Africa	2017–2018	Emergency department	Prospective cohort	HIV, HIV new diagnosis and ART compliance. Questionnaire on HIV treatment adherence, rapid HIV diagnostic test and ELISA	11 383	Median: 36 (IQR: 31–44)	653 (54.2)
Fiseha et al., 2021^71^	Ethiopia	2020	Medical ward	Cross sectional	Hypertension, HIV, diabetes mellitus, chronic kidney disease and smoking. Chronic kidney disease: single creatinine measurement defined as estimated glomerular filtration rate < 60.^c^ Patients with acute kidney injury excluded	369	Mean: 49 (SD: 18)	192 (52.0)
Burke et al., 2021^56^	Malawi	2012–2019	Medical ward	Retrospective cohort	HIV. Electronic medical record database capture	32 814	NR	16 618 (50.6)
Agazhe et al., 2021^57^	Ethiopia	2017–2019	Medical ward	Retrospective cohort	Stroke. Stroke: 62 patients diagnosed with imaging (53 with CT and 9 with MRI) and 33 diagnosed clinically without imaging	3016	NR	NR
Rao et al., 2020^72^	South Africa	2017	Emergency department	Cross sectional	HIV: HIV new diagnosis and HIV status not known (at admission and discharge). Two HIV rapid tests	1880	Median: 33 (IQR: 24–59)	825 (47.4)
Nkoke et al.,2020^73^	Cameroon	2018–2019	Medical ward	Cross sectional	Hypertension emergency. Average of two blood pressure measurements. Hypertensive crisis: SBP/DBP ≥ 180/110	1536	NR	NR
Mulugeta et al., 2020^74^	Ethiopia	2017–2019	Medical ward	Cross sectional	Stroke. Clinical diagnosis with imaging (CT or MRI)	2100	NR	NR
Hertz et al., 2020^38^	United Republic of Tanzania	2019–2019	Emergency department	Prospective cohort	Acute coronary syndrome. Patients with chest pain or shortness of breath had ECG and single troponin	6083	NR	NR
Gilbert et al., 2020^39^	Zimbabwe	2018–2019	Medical ward	Prospective cohort	Hypertension, HIV, diabetes mellitus, heart failure, chronic kidney disease and renal impairment. Chronic kidney disease, hypertension, diabetes and HIV diagnoses from past medical history	253	Mean: 48	137 (54.2)
Du Plooy et al., 2020^40^	South Africa	2013–2014	Medical ward	Prospective cohort	HIV. HIV: medical records and laboratory CD4 T-lymphocytes count	808	Median: 51 (IQR: 36– 65)	534 (52.0)
Woyessa et al., 2019^75^	Ethiopia	2017	Emergency department	Cross sectional	Hypertension emergency, stroke, diabetes mellitus, diabetic emergency, heart failure and chronic obstructive pulmonary disease. No information provided for assessment	889	Mean: 35 (SD: 15)	386 (43.4)
Sheikh et al.,2019^76^	Botswana	2016	Medical ward	Cross sectional	Renal impairment Review of medical charts and serum creatinine results. Renal impairment: estimated glomerular filtration rate < 60^c^	804	NR	NR
Shitandi et al., 2019^41^	Kenya	2015–2016	Medical ward	Prospective cohort	Stroke. Stroke: based on WHO definition with aid of CT and/or MRI imaging	3200	NR	NR
Nkoke et al., 2019^77^	Cameroon	2016–2017	Medical ward	Cross sectional	Hypertension emergency, stroke, acute coronary syndrome and heart failure. All diagnoses ascertained from medical notes	3140	NR	NR
Nakalema et al., 2019^78^	Uganda	2015–2016	Medical ward^ b^	Cross sectional	Hypertension emergency. Average of two blood pressure recordings. Hypertensive crises: SBP/DBP ≥ 180/110	4000	NR	NR
Mwenda et al., 2019^79^	Kenya	2018	Medical ward	Cross sectional	Hypertension, alcohol use, smoking, HIV, diabetes mellitus and chronic kidney disease. Renal impairment: estimated glomerular filtration rate < 60^c^ chronic kidney disease: estimated glomerular filtration rate < 60, with markers of chronic renal damage (laboratory, ultrasound or history of chronic kidney disease > 3 months)	306	Median: 40	144 (47.1)
Mocumbi et al., 2019^80^	Mozambique	2016–2017	Emergency department	Cross sectional	Hypertension emergency, HIV, diabetes mellitus and smoking All diagnoses. ascertained from medical notes	4100	Mean: 37 (SD: 15)	2049 (50.0)
Mandi et al., 2019^42^	Burkina Faso	2016	Medical ward^ b^	Prospective cohort	Hypertension emergency. Average of two blood pressure recordings. Hypertensive crisis: SBP/DBP ≥ 180/120 mmHg	1254	NR	NR
Lakoh et al., 2019^43^	Sierra Leone	2017	Medical ward	Prospective cohort	HIV and HIV new diagnosis. Rapid test for HIV	402	NR	NR
Kalyesubula et al., 2019^81^	Uganda	2011–2014	Medical ward	Cross sectional	Hypertension emergency, HIV, stroke, diabetic emergency, heart failure, alcohol use and renal impairment. Electronic database capture of physician documented diagnoses. Diagnoses base on blood tests, ultrasound, X-ray, ECG, and echocardiography	50 624	Median: 38	26 175 (51.7)
Hertz et al., 2019^58^	United Republic of Tanzania	2017–2018	Emergency department	Retrospective cohort	Hypertension, hypertension emergency, diabetes mellitus and diabetic emergency. Physician documented diagnoses, or any of: hypertension: SBP/DBP ≥ 140/90 mmHg, uncontrolled hypertension: SBP/DBP ≥ 160/100 mmHg, diabetes: random glucose ≥ 200 mg/dL, uncontrolled diabetes mellitus: diabetic ketoacidosis or hyperosmolar hyperglycaemic state or hyperglycaemia > 250 mg/dL	3961	Median: 50 (IQR: 32–67)	2194 (55.4)
Hertz et al., 2019^59^	United Republic of Tanzania	2017–2018	Medical ward^ b^	Retrospective cohort	Stroke, acute coronary syndrome, heart failure and renal impairment. Physician documented diagnoses, supported by laboratory serum analysis (including troponin), imaging (CT and X-ray), ECG, echocardiography	2418	Median: 52	1328 (54.9)
Hansoti et al., 2019^82^	South Africa	2016–2016	Emergency department	Cross sectional	HIV and HIV treatment failure. HIV: laboratory serum analysis for HIV and viral load. HIV treatment failure > 1 000 copies/mL^3^	2100	NR	NR
Hansoti et al., 2019^44^	South Africa	2017–2018	Emergency department	Prospective cohort	HIV, HIV treatment failure, HIV new diagnosis and HIV status not known (at discharge). HIV: laboratory serum analysis for HIV and viral load. HIV treatment failure > 1 000 copies/mL^3^	3537	NR	1123 (38.7)
Haachambwa et al., 2019^45^	Zambia	2017–2018	Medical ward^ b^	Prospective cohort	HIV, HIV treatment failure, HIV new diagnosis and HIV status not known (at discharge). HIV history or dried spot HIV viral load testing. HIV treatment failure > 1 000 copies/mL^3^	1283	Median: 38 (IQR: 30–48)	657 (51.2)
Mchomvu et al., 2019^83^	United Republic of Tanzania	2016–2017	Emergency department	Cross sectional	Hypertension emergency, diabetic emergency, heart failure and renal impairment. Clinician diagnoses. supported by ECG, imaging, blood, urine dip, and echocardiography	23 156	NR	NR
Barak et al., 2019^84^	Botswana	2015–2017	Medical ward	Cross sectional	Hypertension, HIV treatment failure, HIV, HIV new diagnosis, HIV status not known (admission and discharge), stroke, diabetes mellitus and heart failure. HIV: history or CD4 T-lymphocytes count or HIV viral load testing. HIV treatment failure: > 400 copies/mL^3^. Other diagnoses ascertained from clinical notes	2316	Median: 51 (IQR: 34–71)	1237 (53.4)
Shao et al., 2018^46^	United Republic of Tanzania	2015	Emergency department	Prospective cohort	Hypertension emergency. All adult patients screened with blood pressure measurement. Hypertensive crises: SBP/DBP ≥ 180/110 mmHg	8002	NR	NR
Matoga et al., 2018^47^	Malawi	2011–2012	Medical ward	Prospective cohort	HIV, HIV new diagnosis and HIV status not known (at discharge), heart failure. HIV status: history or HIV testing. Other diagnoses ascertained from clinical notes	2911	Mean: 39 (SD: 17)	1457 (50.1)
Perry et al., 2017^48^	Botswana	2011–2012	Medical ward	Prospective cohort	Hypertension emergency, HIV, HIV new diagnosis, HIV status not known (admission and discharge), stroke, diabetic emergency and heart failure. HIV tests. Other diagnoses ascertained from medical records, supported by laboratory and microbiological tests	972	Mean: 48 (SD: 20)	427 (43.9)
Kingery et al., 2017^49^	United Republic of Tanzania	2014	Medical ward	Prospective cohort	HIV, diabetes mellitus, hypertension, hypertension emergency, renal impairment, heart failure, obesity, alcohol use and smoking. Heart failure: Framingham criteria with echocardiography. All patients offered HIV tests, urine dip, serum creatinine. Smoking, alcohol use, hypertension, diabetes from medical history	588	NR	330 (52.0)
Evans et al., 2017^85^	Malawi	2015	Medical ward	Cross sectional	Hypertension, renal impairment, chronic kidney disease, HIV, stroke, chronic liver disease, diabetes mellitus and heart failure. Chronic kidney disease: estimated glomerular filtration rate < 60 for > 3 months. Creatinine and urine measured every 48 hours. Other diagnoses ascertained from medical history	892	Median: 37 (IQR: 30–52)	392 (43.9)
Allain et al., 2017^86^	Malawi	2013–2014	Medical ward	Cross sectional	Hypertension emergency, HIV, stroke, chronic liver disease, diabetes mellitus emergency, heart failure, alcohol use and renal impairment. All diagnoses ascertained from medical notes	10 191	NR	5071 (49.8)
Peck et al., 2016^50^	United Republic of Tanzania	2013	Medical ward	Prospective cohort	HIV, new HIV, diabetes mellitus, hypertension, alcohol use, chronic kidney disease, renal impairment and smoking. HIV tests. Other diagnoses ascertained from medical notes	637	Mean: 47 (SD: 18)	307 (48.2)
Long et al., 2016^60^	South Africa	2010	Medical ward	Retrospective cohort	Hypertension emergency, HIV, HIV status not known (admission), acute coronary syndrome, diabetic emergency and renal impairment. All diagnoses ascertained from electronic medical notes	1041	Median: 42 (IQR: 32–56)	555 (53.3)
Stone et al., 2015^61^	Kenya	2012	Medical ward	Retrospective cohort	HIV. Diagnoses ascertained from medical notes	956	Mean: 42 (SD: 19)	449 (47.0)
Noor et al., 2015^95^	Sudan	2013–2014	Medical ward	Cross sectional	Stroke and diabetic emergency. All diagnoses ascertained from medical notes	2614	Mean: 52 (SD: 19)	1298 (49.7)
Meintjes et al., 2015^51^	South Africa	2012–2013	Medical ward	Prospective cohort	HIV treatment failure, HIV, HIV new diagnosis and HIV status not known (at discharge). HIV status: patients not known to be positive for HIV were offered test with two rapid tests. HIV treatment failure: viral load > 400 copies/mL^3^	1018	NR	NR
Gizaw et al., 2015^62^	Ethiopia	2010–2013	Medical wards	Retrospective cohort	Diabetes mellitus. Diagnoses ascertained from medical notes	8048	NR	NR
Biney et al., 2015^96^	Ghana	2013	Emergency department	Cross-sectional	HIV and HIV status not known (at discharge). Two HIV rapid diagnostic tests	667	Median: 42 (IQR: 30–59)	299 (44.8)
Ogunmola & Oladosu, 2014^98^	Nigeria	2010–2012	Emergency department	Retrospective cohort	HIV, stroke, hypertension emergency, renal impairment, chronic kidney disease, chronic liver disease, acute coronary syndrome, diabetic emergency, heart failure and chronic obstructive pulmonary disease. All diagnoses extracted from medical notes. Echocardiography performed on 59.5% in 2011, not available in 2010. Stroke: CT available for 3.9%	2922	Mean: 52 (SD: 20)	1243 (42.5)
Kakoma et al., 2014^92^	Democratic Republic of the Congo	2011–2012	Medical wards	Cross-sectional	Diabetes mellitus and diabetic emergency. Diabetes mellitus: medical history. Diabetic ketoacidosis: glucose > 11 mmol/L in addition to ketonuria and glycosuria (no plasma pH available)	1020	NR	NR
SanJoaquin et al., 2013^87^	Malawi	2010–2011	Medical wards	Cross sectional	Hypertension, HIV, HIV status not known (discharge), renal impairment, stroke, chronic liver disease and diabetic emergency. Electronic data capture of primary diagnosis	7103	Mean: 37	(50%)
Eyo et al., 2013^88^	Nigeria	2010	Emergency department	Cross sectional	Stroke Diagnoses ascertained from medical notes	1104	Median: 50	NR
Kendig et al., 2013^89^	Malawi	2012–2013	Medical wards	Cross sectional	HIV, new HIV diagnosis and HIV status not known (at discharge). HIV status reviewed on all patients with HIV testing offered if HIV status unknown or last test > 3 months	2985	NR	1325 (44.4)
Anyanwu et al., 2013^97^	Nigeria	2011–2012	Emergency department	Cross sectional	Diabetic emergency. Diagnoses ascertained from medical notes	1703	Mean: 48 (SD: 14)	744 (43.7)
Wachira et al., 2012^90^	Kenya	2010	Emergency department	Cross sectional	Hypertension emergency. Diagnoses ascertained from medical notes	1321	NR	NR

ART: antiretroviral therapy; CT: computed tomography; DBP: diastolic blood pressure; EEG: electroencephalogram; ELISA: enzyme linked immunosorbent assay; HIV: human immunodeficiency virus; IQR: interquartile range; mmHg: millimetres of mercury; MRI: magnetic resonance imaging; NR: not reported or not representative of unselected hospital population; SBP: systolic blood pressure; SD standard deviation.

^a^ The Modification of Diet in Renal Disease equation, as modified by the criteria set by the Kidney Disease Improving Global Outcomes, is used to calculate the estimated glomerular filtration rate.

^b^ Studies recruited participants with acute medical illnesses in emergency departments and triaged for medical care. We have therefore categorised these studies as prevalence estimates among patients from medical wards.

^c^ The estimated glomerular filtration rate is calculated using the Chronic Kidney Disease Epidemiology Collaboration equation.

Note: The average age and the percentage of females reflect values from the unselected hospital population (either emergency department or medical wards). Where studies have not reported figures representative of the unselected population, we have marked this as NR (not reported or not representative of the unselected emergency department or medical ward population.

### Prevalence of primary conditions

#### HIV

HIV prevalence was reported in 32 studies (Table 2). The pooled prevalence of HIV in medical wards was 36.4% (95% CI: 31.3–41.8; 25 studies)^39^^,^^40^^,^^43^^,^^45^^,^^47^^–^^49^^,^^51^^,^^52^^,^^55^^,^^56^^,^^60^^,^^61^^,^^64^^,^^69^^–^^71^^,^^79^^,^^81^^,^^84^^–^^87^^,^^89^ higher than the prevalence in emergency departments (21.9%; 95% CI: 14.5–31.7; seven studies).^37^^,^^44^^,^^65^^,^^72^^,^^80^^,^^82^^,^^96^ HIV infection was reported using laboratory or point-of-care diagnostics in 18 studies,^37^^,^^40^^,^^43^^–^^45^^,^^47^^–^^51^^,^^56^^,^^65^^,^^68^^,^^72^^,^^82^^,^^84^^,^^89^^,^^96^ and by medical records or clinical history in the remaining studies (Table 1).

**Table 2:** Prevalence data of chronic health conditions and risk factors in patients admitted to medical wards or emergency departments, sub-Saharan Africa

Condition, by patient population	No. of patients (no. of studies)	Prevalence, % (range)	95% CI	95% PI	Between group heterogeneity, *P*
**Patients in medical wards**					
Primary preventive conditions					
HIV	231 032 (25)	36.4 (11.4–67.0)	31.3–41.8	14.6–65.8	0.01
Treatment failure	1 037 (3)	31.2 (24.5–45.2)	19.2–46.5	–	0.56
Unknown status on admission	4 329 (3)	37.4 (35.2–41.3)	34.1–40.8	–	< 0.0001
New diagnosis	12 524 (8)	8.8 (4.3–17.2)	4.3–17.2	0.6–62.6	0.20
Unknown status at discharge	19 011 (8)	16.8 (0.5–53.2)	12.3–22.7	5.0–43.7	0.05
Hypertension	122 108 (14)	24.4 (4.1–71.1)	16.7–34.2	4.1–71.1	0.20
Hypertensive emergency	85 333 (11)	5.2 (1.4–14.2)	2.9–8.9	0.5–36.1	0.89
Diabetes	129 627 (15)	11.9 (3.8–25.2)	9.9–14.3	5.4–24.3	0.41
Diabetic emergency	73 988 (8)	4.8 (1.1–17.9)	2.7–8.4	0.6–31.8	0.54
Secondary end-organ conditions					
Heart failure	76 509 (13)	8.2 (2.3–34.2)	5.6–11.9	1.6–33.1	0.34
Stroke	105 506 (17)	6.8 (1.6–32.9)	4.7–9.6	1.3–29.5	0.56
Acute coronary syndrome	11 483 (4)	1.0 (0.1–12.2)	0.2–6.0	–	0.75
Chronic kidney disease	5 408 (8)	7.7 (0.7–38.6)	3.9–14.7	0.6–54.3	0.05
Chronic liver disease	20 550 (6)	2.8 (1.3–5.0)	1.8–4.3	0.6–12.3	0.73
Chronic obstructive pulmonary disease	1 010 (1)	2.0 (2.0–2.0)	1.2–3.0	–	0.20
Risk factors					
Alcohol use	1 960 (5)	21.8 (7.8–51.0)	8.0–47.1	0.3–96.5	–
Smoking	2 686 (7)	9.6 (4.0–31.4)	5.0–17.7	0.8–57.3	0.14
Obesity	588 (1)	10.4 (0.10)	8.0–13.1	–	–
Dyslipidaemia	0 (0)	–	–	–	–
**Patients in emergency departments**					
Primary preventive conditions					
HIV	23 050 (7)	21.9 (6.8–30.2)	14.5–31.7	4.2–64.4	0.01
Treatment failure	463 (2)	25.4 (19.4–32.8)	14.6–40.6	–	0.56
Unknown status on admission	1 880 (1)	75.3 (75.3–75.3)	73.3–77.2	–	< 0.0001
New diagnosis	27 285 (5)	5.0 (3.0–8.1)	3.0–8.1	0.6–29.7	0.20
Unknown status at discharge	7 332 (4)	27.6 (18.0–40.7)	18.5–39.0	–	0.05
Hypertension	8 061 (2)	31.5 (28.9–34.3)	26.4–37.1	–	0.20
Hypertensive emergency	40 251 (6)	5.5 (2.5–14.5)	3.1–9.5	0.6–35.0	0.89
Diabetes	8 950 (3)	9.3 (5.5–13.1)	5.1–16.2	–	0.41
Diabetic emergency	32 631 (5)	3.4 (0.6–6.7)	1.3–8.7	0.1–64.5	0.54
Secondary end-organ conditions					
Heart failure	26 967 (3)	4.0 (1.7–11.3)	0.9–16.1	–	0.34
Stroke	19 359 (5)	5.4 (2.2–13.5)	2.8–10.2	0.4–45.4	0.56
Acute coronary syndrome	9 005 (2)	0.5 (0.1–2.5)	0.0–11.2	–	0.75
Chronic kidney disease	2 922 (1)	3.7 (3.7–3.7)	3.1–4.5	–	0.05
Chronic liver disease	2 922 (1)	3.1 (3.1–3.1)	2.5–3.8	–	0.73
Chronic obstructive pulmonary disease	3 811 (2)	1.0 (0.6–1.5)	0.4–2.6	–	0.20
Risk factors					
Alcohol use	0 (0)	–	–	–	–
Smoking	4 100 (1)	14.8 (14.8–14.8)	13.7–15.9	–	0.14
Obesity	0 (0)	–	–	–	–
Dyslipidaemia	0 (0)	–	–	–	–

Note: We calculated between group heterogeneity using a random effects meta-analysis model, which compares prevalence in medical wards to prevalence in emergency departments. Some conditions have no 95% PI because prediction intervals were calculated when ≥ 5 prevalence estimates were included in the meta-analysis.

Due to limited emergency department data (< 10 studies), only data from medical wards were included in the meta-regression and sub-group analyses. HIV infection prevalence among medical in-patients correlated with national HIV prevalence (odds ratio, OR: 1.33; 95% CI: 1.09–1.63; online repository).^20^ Higher HIV prevalence was noted in southern Africa (46.0%; 95% CI: 40.5–51.7), as compared to eastern Africa (22.5%; 95% CI: 19.8–25.4). There was no association between HIV prevalence and year of study (OR: 0.93; 95% CI: 0.84–1.04); or country-level income status, sex or average age (online repository).^20^ Prevalence of HIV was also unaffected by the removal of studies with a very high risk of bias (36.9%; 95% CI: 31.6–42.6).

**Fig. 2:**
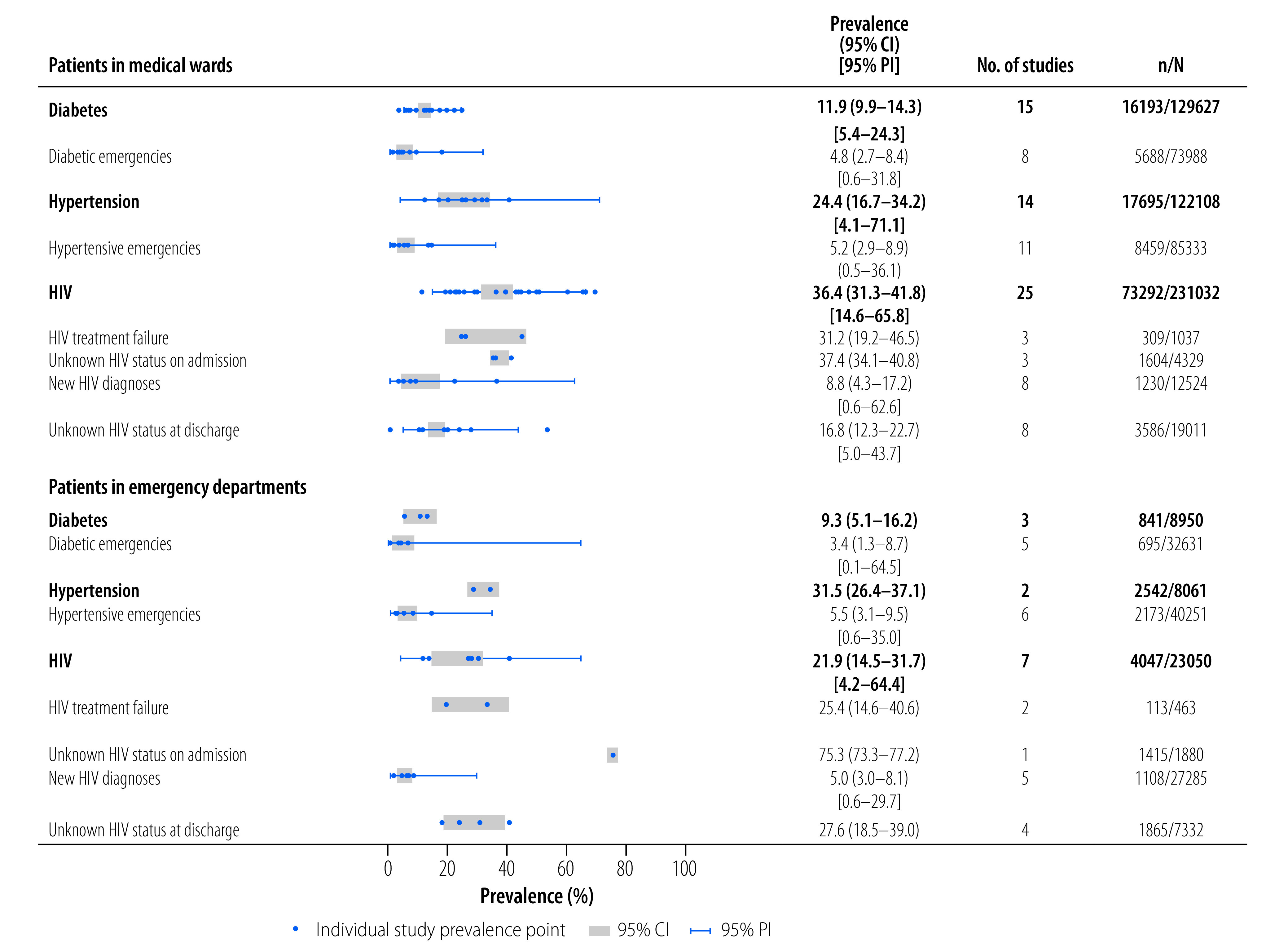
Prevalence of primary preventive chronic conditions in patients admitted to medical wards or emergency departments, sub-Saharan Africa

CI: confidence interval; PI: prediction interval.

Note: Values are also presented in Table 2. Some conditions have no 95% PI because prediction intervals were calculated when ≥ 5 prevalence estimates were included in the meta-analysis

Previously undiagnosed HIV was reported in 13 studies, with a pooled prevalence of 8.8% (95% CI: 4.3–17.2; eight studies)^43^^,^^45^^,^^47^^,^^48^^,^^50^^,^^51^^,^^84^^,^^89^ among medical patients, and 5.0% (95% CI:3.0–8.1; five studies)^37^^,^^44^^,^^65^^,^^68^^,^^72^ among emergency department patients (Fig. 2). Among patients established on antiretroviral therapy, the pooled prevalence of treatment failure was 31.2% (95% CI: 19.2–46.5; three studies)^45^^,^^51^^,^^84^ among medical patients and 25.4% (95% CI: 14.6–40.6; two studies)^44^^,^^82^ among emergency department patients.

#### Hypertension

We estimated the prevalence of hypertension from 16 studies. Pooled prevalence was 24.4% (95% CI: 16.7–34.2; 14 studies)^39^^,^^49^^,^^50^^,^^52^^,^^55^^,^^64^^,^^66^^,^^67^^,^^69^^–^^71^^,^^79^^,^^84^^,^^85^ in medical wards, and 31.5% (95% CI: 26.4–37.1; two studies)^58^^,^^80^ in emergency departments. A high degree of heterogeneity in prevalence estimates was observed in the medical setting (95% PI: 4.1–71.1; *I^2^*: 99.7%). Hypertension diagnoses were classified according to in-patient assessment of blood pressure in two studies (≥ 140/90 mmHg), and from medical records alone in 14 studies (Table 1).

Hypertension prevalence correlated positively with country-level economic status (OR: 1.50; 95% CI: 1.12–2.00; online repository).^20^ We did not find evidence that hypertension prevalence varied by age, sex, study region, study year or study quality (online repository).^20^

Acute hypertensive presentations to hospital were reported in 17 studies, with a pooled prevalence of 5.2% (95% CI: 2.9–8.9; 11 studies)^42^^,^^48^^,^^49^^,^^60^^,^^70^^,^^73^^,^^77^^,^^78^^,^^81^^,^^86^^,^^87^ among medical settings and 5.5% (95% CI: 3.1–9.5; six studies)^46^^,^^58^^,^^75^^,^^83^^,^^90^^,^^98^ in emergency departments.

#### Diabetes

The pooled prevalence of diabetes in medical settings was 11.9% (95% CI: 9.9–14.3; 15 studies; Table 2).^39^^,^^49^^,^^50^^,^^52^^,^^53^^,^^62^^,^^64^^,^^66^^,^^69^^–^^71^^,^^79^^,^^84^^,^^85^^,^^92^ In emergency departments, we found an overlapping prevalence estimate of 9.3% (95% CI: 5.1–16.2; three studies).^58^^,^^75^^,^^80^ A high degree of heterogeneity of estimates in both settings is noted (medical wards 95% PI: 5.4–24.3; *I^2^*: 97.6% and emergency departments *I^2^*: 98.5%; < 5 studies). The heterogeneity within the medical wards could not be explained by differences in age, sex, study quality or study region (online repository).^20^ Diabetes was classified using random glucose measurement (≥ 200 mg/dL) in one study,^58^ and ascertained from medical notes in all other studies (Table 1). Diabetic emergencies were observed in 4.8% (95% CI: 2.7–8.4; eight studies)^48^^,^^60^^,^^81^^,^^86^^,^^87^^,^^92^^,^^94^^,^^95^ of medical patients and 3.4% (95% CI: 1.3–8.7; five studies)^58^^,^^75^^,^^83^^,^^97^^,^^98^ of emergency department patients (Fig. 2).

### Prevalence of secondary conditions

**Fig. 3:**
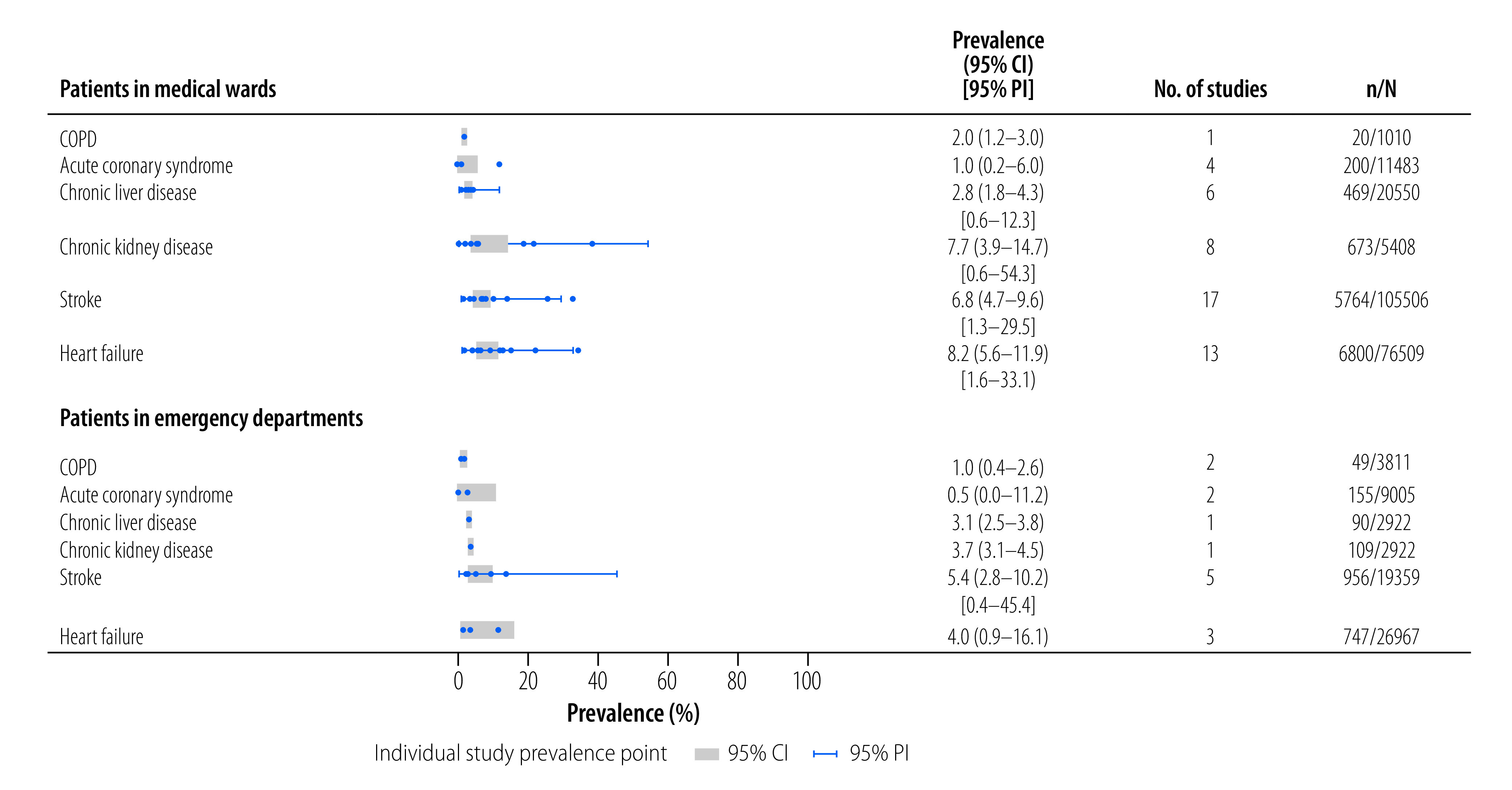
Prevalence of secondary end-organ conditions in patients admitted to medical wards or emergency departments, sub-Saharan Africa

CI: confidence interval; PI: prediction interval.

Note: Values are also presented in Table 2. Some conditions have no 95% PI because prediction intervals were calculated when ≥ 5 prevalence estimates were included in the meta-analysis

Heart failure presentations affected 8.2% (95% CI: 5.6–11.9; 13 studies)^39^^,^^47^^–^^49^^,^^53^^,^^55^^,^^59^^,^^69^^,^^73^^,^^81^^,^^84^^–^^86^ of medical patients, and 4.0% (95% CI: 0.9–16.1; three studies)^75^^,^^83^^,^^98^ of emergency department patients (Fig. 3). Only 31.2% of studies reporting on heart failure described the use of echocardiography.^49^^,^^59^^,^^63^^,^^81^^,^^83^ None of the studies reported prevalence of ischaemic heart disease. However, acute coronary syndrome was found in 1.0% (95% CI: 0.2–6.0; four studies)^59^^,^^60^^,^^70^^,^^73^ of medical patients, and 0.5% (95% CI: 0.0–11.2; two studies)^38^^,^^98^ of emergency department patients. Two studies used electrocardiogram and troponin criteria in making the diagnosis.^38^^,^^59^

Acute exacerbation of chronic obstructive pulmonary disease was observed in 2.0% (95% CI: 1.2–3.0; one study)^69^ of medical patients, and 1.0% (95% CI: 0.4–2.6; two studies)^75^^,^^98^ of emergency department patients. No studies described the use of spirometry to classify diagnoses.

The prevalence of stroke admissions was 6.8% (95% CI: 4.7–9.6; 17 studies)^41^^,^^48^^,^^53^^,^^55^^,^^57^^,^^59^^,^^70^^,^^73^^,^^74^^,^^81^^,^^84^^–^^87^^,^^91^^,^^94^^,^^95^in medical settings, and 5.4% (95% CI: 2.8–10.2; five studies)^54^^,^^75^^,^^88^^,^^93^^,^^98^ in emergency departments. Of these 22 studies, eight used radiological imaging to confirm the diagnosis.^41^^,^^54^^,^^57^^,^^59^^,^^74^^,^^91^^,^^93^^,^^98^

Nine studies reported prevalence of background chronic kidney disease in isolation. Pooled prevalence was 7.7% (95% CI: 3.9–14.7; eight studies)^39^^,^^50^^,^^53^^,^^55^^,^^69^^,^^71^^,^^79^^,^^85^ in medical patients, and 3.7% (95% CI: 3.1–4.5; one study)^98^ in the sole emergency department study. However, only two studies confirmed chronicity using serum and/or sonographic markers of kidney disease.^79^^,^^85^

### Multimorbidity

None of the selected studies investigated multimorbidity prevalence per se. Fourteen studies reported comorbid chronic diseases alongside a primary condition (online repository).^20^ Hypertension and diabetes co-prevalence was reported in three studies: 3790/108 357 (3.5%);^64^ 33/353 (9.3%);^66^ and 273/3961 (6.9%).^58^ HIV and hypertension co-prevalence was reported in three studies: 665/108 357 (0.6%);^64^ 6/972 (0.6%);^48^ and 140/2316 (6.0%).^84^ HIV and diabetes co-prevalence was also reported in three studies: 666/108 358 (0.6%);^64^ 78/2316 (3.4%);^84^ and 78/972 (8.0%).^48^

### Reporting bias

We found evidence of publication bias from small studies reporting prevalence of hypertension (Egger’s *P* -value: 0.04), but no evidence of publication bias for other conditions (online repository).^20^ To reduce very high risk of bias, we conducted sensitivity analyses, and eight studies with very high risk of bias were subsequently excluded from the meta-analyses, with no observed changes in synthesized prevalence estimates (online repository).^20^

## Discussion

Here we present synthesized prevalence data for multiple individual chronic diseases among hospitalized adults in sub-Saharan Africa. We found no studies that directly measured the prevalence of multimorbidity, although secondary analyses within these studies suggest this to be a major problem. HIV, hypertension and diabetes were all common, mirrored by high proportions of patients presenting with decompensated disease. These presentations included hypertensive or diabetic emergencies; undiagnosed or undertreated HIV; and end-organ secondary complications such as heart failure, chronic kidney disease and stroke. Improvements in early recognition and management of chronic diseases are likely to result in improved healthy life expectancy for the most vulnerable patients.

Our estimated HIV prevalence in sub-Saharan African hospitals is about eightfold higher than reported estimates at the community level in sub-Saharan Africa (36.4% versus 4.7%).^14^ Reassuringly, more than 90% of HIV-infected patients included in this review knew their diagnosis; however, treatment failure in about one third of patients indicates that viral control should be a keystone issue for future public health campaigns. Absence of temporal changes in our review may reflect regional and sub-national variability in the HIV epidemic and ART scale-up.^8^^,^^34^ This result contrasts data from Malawi which shows falling HIV admissions from 2012 to 2019.^56^

For hypertension, prevalence in sub-Saharan communities is estimated at 30.8%,^99^ which is similar to hospital prevalence found in our study (24.4%). We found that admission with severe uncontrolled hypertension was higher than in high-income countries (5.2% versus 1.9%).^100^ With diabetes, the estimated hospital prevalence was 11.9% which is higher than community levels in sub-Saharan Africa (4.2%).^14^ Diabetic emergencies represented 40% of patients admitted with diabetes. In contrast, findings from the National Diabetes Inpatient Audit England 2019 found that diabetic emergencies were approximately one in 20 in diabetic inpatients.^101^ In sub-Saharan hospitals, the high burden of decompensated illness presentations indicate missed opportunities to better diagnose and control disease.

A similar pattern was seen in disease burden from end-organ complications, dominated by heart failure and stroke (8.2% and 6.8%, respectively), which are higher than estimates from outside sub-Saharan Africa (1–2%^102^^,^^103^ and 3.7–4.4%).^104^^,^^105^ This reinforces observations that in sub-Saharan Africa: (i) hypertension is the leading cause of heart failure and stroke; and (ii) 88% of the global hypertension mortality is found in low- and middle-income countries.^106^ Estimates from the Global Burden of Disease 2017 suggest that ischaemic heart disease is the most common cause of cardiovascular-related death in sub-Saharan Africa (5% of all deaths).^107^ Although we did not find data on the prevalence of ischaemic heart disease, our result on prevalence of acute coronary syndromes (1%) suggests low rates of ischaemic heart disease in sub-Saharan hospitals, in line with previous hospital-based observations.^108^^–^^111^

The strengths of our study include studies reporting data from unselected medical and emergency department populations, designed to reduce selection bias. There were no language restrictions in our search strategy, and we were able to include data from 20 sub-Saharan African countries, representative of nearly 400 000 patient admissions. We explored heterogeneity by calculating 95% prediction intervals to provide clinically relevant information on the degree of heterogeneiety.^30^ In addition, we restricted our pooled synthesis and used robust methods to explore potential explanations, including predetermined sensitivity, subgroup, and meta-regression analysis.

The heterogeneity observed in our analysis is a common limitation of systematic reviews of disease prevalence.^7^^,^^112^ The reasons for this include differences in population demographics; criteria and tools used to ascertain outcomes; and study quality. Given differences between countries in terms of access to health care, socioeconomic status, geography and ethnicity, heterogeneity both between and within countries in sub-Saharan Africa is expected. For example, HIV prevalence is likely to be higher in hospitalized patients compared to the general population for a given country, with multiple factors (e.g. success in meeting the UNAIDS 90–90–90 objectives)^113^ influencing this relationship. 

Another limitation was the non-uniform application of diagnostic criteria, and likely inconsistent access to laboratory assays, equipment and technical expertise. Quality issues relating to outcome ascertainment were identified in over half of all included studies. For instance, troponin, electrocardiogram and angiography were underutilized in the diagnosis of acute coronary syndrome, and spirometry for chronic obstructive pulmonary disease. This underutilization may have led to underreporting of these conditions. Although 13.1% of studies were at very high risk of bias, sensitivity analyses demonstrated consistent disease prevalence estimates.

A key finding from this systematic review is the lack of primary outcome data on multimorbidity in sub-Saharan hospitals. Synthesized community-level data from predominantly high-income settings have estimated multimorbidity prevalence is 33.1%,^114^ with disease combinations reflecting the most prevalent individual long-term conditions within the population.^115^ Prospective cohort studies, designed explicitly to examine multimorbidity prevalence using standardized diagnostic tools and criteria, could support the development of health services more responsive to patient need. 

We found high prevalence of single chronic diseases in hospital settings. From the limited data on multimorbidity identified within the secondary analyses of included studies, it is probable that there is a high burden of missed multimorbidity in sub-Saharan Africa. When examining the secondary outcome data from the studies included in this analysis, it was revealed that there may be a significant burden of multimorbidity in this particular context. For instance, one study primarily focused on investigating the prevalence of hypertension among medical in-patients, but it also discovered that out of the 59 patients with hypertension, 33 of them were also diagnosed with diabetes.^66^ We also found high prevalence of acute decompensated presentations. We observed increased chronic disease prevalence within hospitals compared to community settings. Hospitalized patients in sub-Saharan Africa are therefore likely to have increased preventable disability and early mortality compared to high-income settings.

Our review suggests important clinical and policy implications. Similarly, the need for context-appropriate diagnostics was underscored by a 2023 World Health Assembly resolution.^116^ Inconsistent use of diagnostic tools and criteria has also been described within the *Lancet* commission on diagnostics, showing limited or no access for 47% of the global population.^117^ Implementation of standardized chronic disease programmes which focus on community care (e.g. the WHO package of noncommunicable disease interventions)^118^ could be strengthened by explicit linkages to secondary clinical pathways. 

Successful implementation of such linkages will require broad health systems approaches including: health-care worker training; development of financial models that promote reliable access to diagnostics and essential medicines; integration with existing health information systems;^119^ robust governance structures; and strengthened local leadership.^116^ The need to shift away from disease- to patient- centred approaches is a consistent theme highlighted in recent *Lancet* commissions.^120^^,^^121^ Improved health literacy is likely to empower patients and their caregivers in managing their health and chronic conditions, and navigating care pathways. Policies which link primary and secondary care for chronic disease management could facilitate more accessible and cost-effective models of care delivery, from both provider and patient perspectives.
